# Supplementary material for: An experimental investigation of Lean Six Sigma philosophies in a high-mix low-volume manufacturing environment
Source: PLoS One. 2024 May 17;19(5):e0299498. doi: 10.1371/journal.pone.0299498 (PMC11101027; doi:10.1371/journal.pone.0299498)
Supplement: S1 Appendix — (DOCX) [file pone.0299498.s001.docx]

# 8. Appendix A: DMAIC For Experimental Approaches

For the experimental interventions described in this article, the format of Define, Measure, Analyze, Improve, Control (DMAIC) was used [21] This approach is typical in the application of Lean Six Sigma philosophies and provides advantages in addressing the root causes of inefficient or wasteful processes [22]. The application of each phase of the DMAIC processes as it was used for the experimental interventions is described below.

## 8.1. Define

The define phase is used to detail the problem that needs to be addressed. For both experimental interventions, it was determined that the problem was production flow. As this was addressed, it was found that there were multiple contributors to inhibited production flow and, as a result, overall cost to manufacture components. These included the quality of components (including the need for rework when components were not correct the first time), the amount of space being used on the shop floor to produce the components, and the throughput of components being manufactured.

## 8.2. Measure

The measure phase is used to determine which measurements are appropriate to ensure that the intended outcome is achieved. For each experimental intervention, the baseline of an unaltered production environment was used. The specific variables used are detailed in Table 3. Table 4 addresses how these variables were used to calculate the metrics.

## 8.3. Analyze

The analyze phase is used to determine which of the variables provides the best opportunity for improvement of the overall process. For the experimental interventions, an overall focus on production flow was used. By focusing on overall flow, there would be impacts to the largest number of metrics.

## 8.4. Improve

The improve phase is used to reduce the causes of waste in the production process. For the first experimental intervention, the implementation of cellular flow, the production flow was changed to reduce the wait time between operations, reduce the required floorspace by reducing the number of WIP areas needed, and move “non-value added” activities to be internal to “value-added” activities.

For the second intervention, the implementation of single machine processing, the production flow was changed to reduce the number of machines used to complete the components from three machines to one. This intervention eliminated the wait time between operations and reduced the required floor space needed to produce the components.

## 8.5. Control

The control phase is used to maintain the improvements that were made. Considering the experimental treatments used and the resulting metrics, the cellular process flow remained in place in the production environment. Related documentation, such as work instructions, and production routings, were updated to define this new process as the standard process.
