# Supplementary material for: An experimental investigation of Lean Six Sigma philosophies in a high-mix low-volume manufacturing environment
Source: PLoS One. 2024 May 17;19(5):e0299498. doi: 10.1371/journal.pone.0299498 (PMC11101027; doi:10.1371/journal.pone.0299498)
Supplement: S2 Appendix — (DOCX) [file pone.0299498.s002.docx]

# 9. Appendix B: Non-stationarity of production statistics under HMLV manufacturing

Fig 8: Lead Time (Hours) Compared to Completion Dates per Batch for Baseline and Experimental Treatments

Within the baseline results, we can observe substantial variation between lead time and completion date. This variation reflects the constantly shifting priorities and intricacies of value streams in the HMLV setting, even under baseline conditions. As shown in Fig 8, there is an absence of a clear dependency between lead time and completion date for both the baseline and the experimental treatments. Outliers such as those shown in Fig 8 are not the results of aleatory uncertainty, these were orders created for components to build safety stock, but they are not prioritized because they were not immediately needed. This type of demand unpredictability is common in HMLV environments and leads to non-stationarity of distributions of measurands in HMLV manufacturing.
