# Supplementary material for: An experimental investigation of Lean Six Sigma philosophies in a high-mix low-volume manufacturing environment
Source: PLoS One. 2024 May 17;19(5):e0299498. doi: 10.1371/journal.pone.0299498 (PMC11101027; doi:10.1371/journal.pone.0299498)
Supplement: S3 Appendix — (DOCX) [file pone.0299498.s003.docx]

# 10. Appendix C: Variables and Dataset

| **Variable** | **Definition** | **Baseline** | **Cellular** | **Single WC** |
| --- | --- | --- | --- | --- |
| **C_S_** | Cost of space | $112,500.00 | $60,000.00 | $37,500.00 |
| **D** | Defects | 0.12% | 0.10% | 0.26% |
| **IW** | Inventory value in WIP | $73,193.99 | $11,820.80 | $44,609.36 |
| **M_S_** | Machine Setup Time | 3.38 | 2.84 | 6.11 |
| **OH_EX_** | Overhead multiplier for the experiment period | 0.10 | 0.10 | 0.10 |
| **OH_WC_** | Overhead for the parts produced (3 months) | $77.23 | $77.23 | $77.23 |
| **OW_WC_** | Operator wage | $26.25 | $26.25 | $26.25 |
| **Q_B_** | Quantity of components in batch | 58 | 58 | 58 |
| **Q_S_** | Quantity of components scrapped in batch | 0.07 | 0.06 | 0.15 |
| **σ** | Standard Deviation | 851.14 | 361.73 | 412.02 |
| **T_C_** | Cycle Time (per part) | 0.59 | 0.59 | 1.63 |
| **T_J_** | Time operator is on specific job | 11.97 | 9.85 | 47.49 |
| **T_O_** | Labor time for operator | 11.97 | 9.85 | 29.34 |
| **T_P_** | Planned production time | 9.86 | 8.14 | 9.44 |
| **X-bar** | Mean or average change in process over time | 768.83 | 548.84 | 1047.87 |

| **ID** | **Work Order Number** | **Completion Date** | **Part Number** | **Batch Quantity** | **Value in WIP (USD not discounted)** | **Lead time (hr)** | **Job Run Time (hr)** | **Cycle Time (hr)** | **Setup Time (hr)** | **Time on Job (hr)** | **Planned Time (hr)** | **Run Labor Standard (hr)** | **Setup Standard (hr)** | **Actual cycle time total per batch run (hr)** |
| --- | --- | --- | --- | --- | --- | --- | --- | --- | --- | --- | --- | --- | --- | --- |
| **Baseline** | 119679 | 7/21/2022 | 5000254 | 50 | 19,653.00 | 1487.71 | 6.53 | 0.13 | 4.08 | 10.61 | 6.26 | 95.24 | 1.50 | 6.53 |
| **Baseline** | 124396 | 7/29/2022 | 5009002 | 20 | 9,997.60 | 0.18 | 3.02 | 0.15 | 0.82 | 3.84 | 7.51 | 300.30 | 1.50 | 3.02 |
| **Baseline** | 124463 | 7/18/2022 | 5003306 | 50 | 21,795.50 | 1487.57 | 6.86 | 0.14 | 2.78 | 9.64 | 8.78 | 135.69 | 2.00 | 6.86 |
| **Baseline** | 124836 | 8/1/2022 | 1183900 | 50 | 6,792.00 | 960.03 | 5.80 | 0.12 | 2.80 | 8.60 | 7.11 | 72.10 | 3.50 | 5.80 |
| **Baseline** | 125136 | 7/6/2022 | 5001213 | 6 | 7,710.06 | 1295.73 | 1.90 | 0.32 | 5.92 | 7.82 | 7.02 | 253.70 | 5.50 | 1.90 |
| **Baseline** | 125658 | 6/23/2022 | 5005900 | 6 | 2,617.08 | 167.75 | 0.85 | 0.14 | 4.24 | 5.09 | 3.60 | 183.33 | 2.50 | 0.85 |
| **Baseline** | 126034 | 7/1/2022 | 5001220 | 12 | 7,639.60 | 144.29 | 5.17 | 0.43 | 9.63 | 14.80 | 8.44 | 245.06 | 5.50 | 5.17 |
| **Baseline** | 126408 | 11/1/2022 | 5021210 | 60 | 33,550.80 | 3863.96 | 32.86 | 0.55 | 9.52 | 42.38 | 34.50 | 508.35 | 4.00 | 32.86 |
| **Baseline** | 126855 | 6/16/2022 | 2700808 | 7 | 3,724.00 | 23.62 | 4.33 | 0.62 | 3.00 | 7.33 | 2.92 | 166.67 | 1.75 | 4.33 |
| **Single WC** | **127430** | 6/17/2022 | 5323904 | 39 | 15,745.47 | 5.05 | 3.35 | 0.09 | 2.25 | 5.60 | 3.06 | 40.00 | 1.50 | 3.35 |
| **Single WC** | **126001** | 6/26/2022 | 2317100 | 12 | 13,214.28 | 96.05 | 6.17 | 0.51 | 6.83 | 13.00 | 2.08 | 90.00 | 1.00 | 6.17 |
| **Single WC** | **119654** | 7/12/2022 | 2050272 | 90 | 30,812.40 | 647.86 | 48.61 | 0.54 | 7.07 | 55.68 | 5.50 | 50.00 | 1.00 | 48.61 |
| **Single WC** | **127254** | 6/29/2022 | 2803619 | 2 | 2,624.24 | 287.84 | 2.35 | 1.18 | 10.06 | 12.41 | 2.20 | 100.67 | 2.00 | 2.35 |
| **Single WC** | **124308** | 6/14/2022 | 2808801 | 30 | 5,181.00 | 24.04 | 2.94 | 0.10 | 3.94 | 6.88 | 3.42 | 60.00 | 1.62 | 2.94 |
| **Single WC** | **123910** | 6/16/2022 | 2310501 | 100 | 23,051.00 | 168.18 | 54.61 | 0.55 | 7.66 | 62.27 | 8.93 | 81.81 | 0.75 | 54.61 |
| **Single WC** | **124586** | 6/27/2022 | 2309814 | 135 | 181,728.90 | 1127.45 | 44.61 | 0.33 | 4.96 | 49.57 | 40.91 | 267.86 | 4.75 | 44.61 |
| **Cellular** | **119679** | 6/13/2022 | 5000254 | 50 | 22,153.00 | 575.79 | 19.67 | 0.39 | 1.75 | 21.42 | 7.38 | 117.65 | 1.50 | 19.67 |
| **Cellular** | **119679** | 6/13/2022 | 5000254 | 50 | 19,653.00 | 575.79 | 6.53 | 0.13 | 4.08 | 10.61 | 6.26 | 95.24 | 1.50 | 6.53 |
| **Cellular** | **124187** | 6/14/2022 | 5014200 | 20 | 35,542.20 | 791.93 | 7.42 | 0.37 | 3.05 | 10.47 | 11.50 | 400.00 | 3.50 | 7.42 |
| **Cellular** | **124396** | 7/29/2022 | 5009002 | 20 | 9,997.60 | 0.18 | 3.02 | 0.15 | 0.82 | 3.84 | 7.51 | 300.30 | 1.50 | 3.02 |
| **Cellular** | **124463** | 7/1/2022 | 5003306 | 50 | 24,595.50 | 1079.57 | 0.92 | 0.02 | 1.75 | 2.67 | 8.20 | 137.93 | 1.30 | 0.92 |
| **Cellular** | **124463** | 7/1/2022 | 5003306 | 50 | 21,795.50 | 1079.57 | 6.86 | 0.14 | 2.78 | 9.64 | 8.78 | 135.69 | 2.00 | 6.86 |
| **Cellular** | **124816** | 5/30/2022 | 5021100 | 25 | 9,030.25 | 479.57 | 8.47 | 0.34 | 2.95 | 11.42 | 5.92 | 166.67 | 1.75 | 8.47 |
| **Cellular** | **124836** | 8/1/2022 | 1183900 | 50 | 6,792.00 | 960.03 | 5.80 | 0.12 | 2.80 | 8.60 | 7.11 | 72.10 | 3.50 | 5.80 |
| **Cellular** | **125095** | 6/16/2022 | 5022101 | 40 | 61,786.00 | 168.28 | 9.50 | 0.24 | 2.47 | 11.97 | 11.73 | 243.24 | 2.00 | 9.50 |
| **Cellular** | **125136** | 6/8/2022 | 5001213 | 6 | 7,710.06 | 623.73 | 1.90 | 0.32 | 5.92 | 7.82 | 7.02 | 253.70 | 5.50 | 1.90 |
| **Cellular** | **125359** | 6/8/2022 | 5017000 | 50 | 17,526.50 | 360.15 | 7.72 | 0.15 | 2.26 | 9.98 | 8.39 | 142.86 | 1.25 | 7.72 |
| **Cellular** | **125588** | 6/27/2022 | 2700603 | 28 | 24,876.04 | 744.21 | 29.80 | 1.06 | 1.05 | 30.85 | 44.88 | 1538.46 | 1.80 | 29.80 |
| **Cellular** | **125658** | 6/23/2022 | 5009500 | 6 | 8,509.08 | 167.75 | 1.33 | 0.22 | 1.16 | 2.49 | 5.45 | 200.00 | 4.25 | 1.33 |
| **Cellular** | **125658** | 6/23/2022 | 5005900 | 6 | 2,617.08 | 167.75 | 0.85 | 0.14 | 4.24 | 5.09 | 3.60 | 183.33 | 2.50 | 0.85 |
| **Cellular** | **125659** | 5/30/2022 | 5008401 | 6 | 6,244.08 | 311.47 | 2.13 | 0.36 | 2.55 | 4.68 | 3.88 | 313.24 | 2.00 | 2.13 |
| **Cellular** | **126034** | 7/1/2022 | 5001220 | 12 | 7,639.60 | 144.29 | 5.17 | 0.43 | 9.63 | 14.80 | 8.44 | 245.06 | 5.50 | 5.17 |
| **Cellular** | **126261** | 6/21/2022 | 2700712 | 6 | 1,888.14 | 119.55 | 8.71 | 1.45 | 3.69 | 12.40 | 4.50 | 500.00 | 1.50 | 8.71 |
| **Cellular** | **126262** | 6/20/2022 | 2700812 | 6 | 3,406.26 | 119.72 | 14.48 | 2.41 | 2.15 | 16.63 | 9.82 | 1428.57 | 1.25 | 14.48 |
| **Cellular** | **126408** | 10/15/2022 | 5021210 | 60 | 33,550.80 | 3455.96 | 32.86 | 0.55 | 9.52 | 42.38 | 34.50 | 508.35 | 4.00 | 32.86 |
| **Cellular** | **126834** | 8/1/2022 | 5016112 | 7 | 15,535.94 | 455.67 | 1.09 | 0.16 | 1.50 | 2.59 | 4.16 | 380.00 | 1.50 | 1.09 |
| **Cellular** | **126855** | 6/16/2022 | 2700808 | 7 | 3,724.00 | 23.62 | 4.33 | 0.62 | 3.00 | 7.33 | 2.92 | 166.67 | 1.75 | 4.33 |
| **Cellular** | **126855** | 6/16/2022 | 2700808 | 15 | 6,930.00 | 23.62 | 15.21 | 1.01 | 1.02 | 16.23 | 22.24 | 1315.79 | 2.50 | 15.21 |
| **Cellular** | **127251** | 7/3/2022 | 2700701 | 6 | 6,692.64 | 360.12 | 4.93 | 0.82 | 1.23 | 6.16 | 1.01 | 1.00 | 1.00 | 4.93 |
| **Cellular** | **127347** | 6/30/2022 | RB4020 | 3 | 936.56 | 383.83 | 7.40 | 2.47 | 9.77 | 17.17 | 1.55 | 181.82 | 1.00 | 7.40 |
