# Supplementary material for: An experimental investigation of Lean Six Sigma philosophies in a high-mix low-volume manufacturing environment
Source: PLoS One. 2024 May 17;19(5):e0299498. doi: 10.1371/journal.pone.0299498 (PMC11101027; doi:10.1371/journal.pone.0299498)
Supplement: S1 Dataset — (PDF) [file pone.0299498.s004.pdf]

Variable	Definition	Single WC	Cellular	Variable
C_S	Cost of space	$37,500.00	$60,000.00	$112,500.00
**D**	Defects	0.26%	0.10%	0.12%
**IW**	Inventory value in WIP	$44,609.36	$11,820.80	$73,193.99
M_S	Machine Setup Time	6.11	2.84	3.38
OH_{EX}	Overhead multiplier for the experiment period	0.10	0.10	0.10
OH_{WC}	Overhead for the parts produced (3 months)	$77.23	$77.23	$77.23
OW_{WC}	Operator wage	$26.25	$26.25	$26.25
Q_B	Quantity of components in batch	58	58	58
Q_S	Quantity of components scrapped in batch	0.15	0.06	0.07
σ	Standard Deviation	412.02	361.73	851.14
T_C	Cycle Time (per part)	1.63	0.59	0.59
T_J	Time operator is on specific job	47.49	9.85	11.97
T_O	Labor time for operator	29.34	9.85	11.97
T_P	Planned production time	9.44	8.14	9.86
**X-bar**	Mean or average change in process over time	1047.87	548.84	768.83

ID	Work Order Number	Part Number	Batch Quantity	Value in WIP (USD not discounted)	Lead time (hr)	Job Run Time (hr)	Cycle Time (hr)	Setup Time (hr)	Time on Job (hr)	Planned Time (hr)	Run Labor Standard (hr)	Setup Standard (hr)	Actual cycle time total per batch run (hr)
**Baseline**	119679	5000254	50	19,653.00	1487.71	6.53	0.13	4.08	10.61	6.26	95.24	1.50	6.53
**Baseline**	124396	5009002	20	9,997.60	0.18	3.02	0.15	0.82	3.84	7.51	300.30	1.50	3.02
**Baseline**	124463	5003306	50	21,795.50	1487.57	6.86	0.14	2.78	9.64	8.78	135.69	2.00	6.86
**Baseline**	124836	1183900	50	6,792.00	960.03	5.80	0.12	2.80	8.60	7.11	72.10	3.50	5.80
**Baseline**	125136	5001213	6	7,710.06	1295.73	1.90	0.32	5.92	7.82	7.02	253.70	5.50	1.90
**Baseline**	125658	5005900	6	2,617.08	167.75	0.85	0.14	4.24	5.09	3.60	183.33	2.50	0.85
**Baseline**	126034	5001220	12	7,639.60	144.29	5.17	0.43	9.63	14.80	8.44	245.06	5.50	5.17
**Baseline**	126408	5021210	60	33,550.80	3863.96	32.86	0.55	9.52	42.38	34.50	508.35	4.00	32.86
**Baseline**	126855	2700808	7	3,724.00	23.62	4.33	0.62	3.00	7.33	2.92	166.67	1.75	4.33
**Single WC**	**127430**	5323904	39	15,745.47	5.05	3.35	0.09	2.25	5.60	3.06	40.00	1.50	3.35
**Single WC**	**126001**	2317100	12	13,214.28	96.05	6.17	0.51	6.83	13.00	2.08	90.00	1.00	6.17
**Single WC**	**119654**	2050272	90	30,812.40	647.86	48.61	0.54	7.07	55.68	5.50	50.00	1.00	48.61
**Single WC**	**127254**	2803619	2	2,624.24	287.84	2.35	1.18	10.06	12.41	2.20	100.67	2.00	2.35
**Single WC**	**124308**	2808801	30	5,181.00	24.04	2.94	0.10	3.94	6.88	3.42	60.00	1.62	2.94
**Single WC**	**123910**	2310501	100	23,051.00	168.18	54.61	0.55	7.66	62.27	8.93	81.81	0.75	54.61
**Single WC**	**124586**	2309814	135	181,728.90	1127.45	44.61	0.33	4.96	49.57	40.91	267.86	4.75	44.61
**Cellular**	**119679**	5000254	50	22,153.00	575.79	19.67	0.39	1.75	21.42	7.38	117.65	1.50	19.67
**Cellular**	**119679**	5000254	50	19,653.00	575.79	6.53	0.13	4.08	10.61	6.26	95.24	1.50	6.53
**Cellular**	**124187**	5014200	20	35,542.20	791.93	7.42	0.37	3.05	10.47	11.50	400.00	3.50	7.42
**Cellular**	**124396**	5009002	20	9,997.60	0.18	3.02	0.15	0.82	3.84	7.51	300.30	1.50	3.02
**Cellular**	**124463**	5003306	50	24,595.50	1079.57	0.92	0.02	1.75	2.67	8.20	137.93	1.30	0.92
**Cellular**	**124463**	5003306	50	21,795.50	1079.57	6.86	0.14	2.78	9.64	8.78	135.69	2.00	6.86
**Cellular**	**124816**	5021100	25	9,030.25	479.57	8.47	0.34	2.95	11.42	5.92	166.67	1.75	8.47
**Cellular**	**124836**	1183900	50	6,792.00	960.03	5.80	0.12	2.80	8.60	7.11	72.10	3.50	5.80
**Cellular**	**125095**	5022101	40	61,786.00	168.28	9.50	0.24	2.47	11.97	11.73	243.24	2.00	9.50
**Cellular**	**125136**	5001213	6	7,710.06	623.73	1.90	0.32	5.92	7.82	7.02	253.70	5.50	1.90
**Cellular**	**125359**	5017000	50	17,526.50	360.15	7.72	0.15	2.26	9.98	8.39	142.86	1.25	7.72
**Cellular**	**125588**	2700603	28	24,876.04	744.21	29.80	1.06	1.05	30.85	44.88	1538.46	1.80	29.80
**Cellular**	**125658**	5009500	6	8,509.08	167.75	1.33	0.22	1.16	2.49	5.45	200.00	4.25	1.33
**Cellular**	**125658**	5005900	6	2,617.08	167.75	0.85	0.14	4.24	5.09	3.60	183.33	2.50	0.85
**Cellular**	**125659**	5008401	6	6,244.08	311.47	2.13	0.36	2.55	4.68	3.88	313.24	2.00	2.13
**Cellular**	**126034**	5001220	12	7,639.60	144.29	5.17	0.43	9.63	14.80	8.44	245.06	5.50	5.17
**Cellular**	**126261**	2700712	6	1,888.14	119.55	8.71	1.45	3.69	12.40	4.50	500.00	1.50	8.71
**Cellular**	**126262**	2700812	6	3,406.26	119.72	14.48	2.41	2.15	16.63	9.82	1428.57	1.25	14.48
**Cellular**	**126408**	5021210	60	33,550.80	3455.96	32.86	0.55	9.52	42.38	34.50	508.35	4.00	32.86
**Cellular**	**126834**	5016112	7	15,535.94	455.67	1.09	0.16	1.50	2.59	4.16	380.00	1.50	1.09
**Cellular**	**126855**	2700808	7	3,724.00	23.62	4.33	0.62	3.00	7.33	2.92	166.67	1.75	4.33
**Cellular**	**126855**	2700808	15	6,930.00	23.62	15.21	1.01	1.02	16.23	22.24	1315.79	2.50	15.21
**Cellular**	**127251**	2700701	6	6,692.64	360.12	4.93	0.82	1.23	6.16	1.01	1.00	1.00	4.93
**Cellular**	**127347**	RB4020	3	936.56	383.83	7.40	2.47	9.77	17.17	1.55	181.82	1.00	7.40
