# Supplementary material for: An experimental investigation of Lean Six Sigma philosophies in a high-mix low-volume manufacturing environment
Source: PLoS One. 2024 May 17;19(5):e0299498. doi: 10.1371/journal.pone.0299498 (PMC11101027; doi:10.1371/journal.pone.0299498)
Supplement: Attachment — Submitted filename: Response to Reviewers.pdf [file pone.0299498.s005.pdf]

Original Manuscript ID: PONE-D-23-20942

Original Article Title: “An experimental investigation of Lean Six Sigma philosophies in a high-mix low-volume manufacturing environment”

To: PLOS ONE Editor

Re: Response to Reviewers

Dear Editor:

Thanks to you and the reviewers for their input and for allowing a resubmission of our manuscript. We believe that we have addressed all the comments from the reviewers, and that we submit a revised and much-improved manuscript.

We are uploading (a) our point-by-point response to the comments (below) (“Response to Reviewers”), (b) an updated manuscript with green highlighting indicating changes (as “Revised Manuscript with Track Changes”), and (c) a clean, updated manuscript without highlights (“Manuscript”).

Thank you,

Amanda Normand and Thomas Bradley

Response to Reviewers

Reviewer #1 Comment Responses

Reviewer #1: This article discusses the impact of three different LSS interventions on HMLV manufacturing. The article is well within the scope of the Journal. However, some shortcomings regarding the text, literature review, sufficient experimental details, and discussion need to be addressed before publication. These are some suggestions to improve the article's quality.

1. **The article lacks a comprehensive literature review on LSS and HMLV manufacturing. It would be beneficial to add a dedicated section for this purpose.**

Author Response: Thank you for this suggestion.

Author Action: We have added a new section after the introduction titled “Literature Review” and revised the content (1.1.). Please find this section highlighted in green in the “Revised Manuscript with Track Changes”.

2. **Experiments data presented is insufficient to conclude the results.**

Author Response: Thank you for your comment.

Author Action: Additional data has been made available under https://doi.org/10.5061/dryad.8pk0p2nvv .

Data will be available there after our libraries’ internal checks and assurances are completed.

Bradley, Thomas; Normand, Amanda (Forthcoming 2024). An experimental investigation of Lean Six Sigma philosophies in a high-mix low-volume manufacturing environment [Dataset]. Dryad.

3. **The inclusion of better pictorial representations of the experiments would enhance reader engagement.**

Author Response: We agree that additional pictorial representations would enhance reader engagement.

Author Action: We have added an additional figure (Figure 4) to better describe the second experimental intervention (single machine processes) to share the same type of pictorial representations as the baseline and intervention 1.

4. **While LSS philosophies typically follow the DMAIC approach, this article does not delve into this aspect. A significant revision is required to improve the overall structure.**

Author Response: Thank you for your comment. This paper is concentrating more on the use of Lean Six Sigma philosophies to improve high-mix low-volume manufacturing environment. We concentrate in this paper on comparing Lean Six Sigma to other methods. Please note that this paper is a set of experiments rather than a case study.

Author Action: Appendix A has been added to discuss the characteristics of the DMAIC approach for this experiment.

5. **Review the sentences in line number 24 and line number 73 for accuracy and clarity.**

Author Response: Thank you for your careful examination of the manuscript.

Author Action: Lines 24 and 73 were updated for accuracy and clarity.

6. **The authors make use of VSM, but they have not provided an explanation of what it is or why it's being used.**

Author Response: We agree that a stronger explanation of what a VSM is and why it is used is needed.

Author Action A definition, including the intended use of VSM's has been added in Section 1.1. Please find these changes highlighted in green in the "Revised Manuscript with Track Changes" document.

7. **The concept of staging events and their impact on process downtime is not adequately explained. This should be addressed for a more comprehensive understanding.**

Author Response: Thank you for this insight.

Author Action: A description of the unique characteristics of HMLV manufacturing that allow for experimentation without significant production disruption has been added to section 2.1. Please find this update highlighted in green in the "Revised Manuscript with Track Changes".

8. **To enhance the quality of this article, it is recommended to refer to the following related sources**: https://doi.org/10.1016/j.heliyon.2022.e09043, https://doi.org/10.1108/IJQRM-08-2022-0250, https://doi.org/10.1080/09537287.2016.1185188

Author Response: Thank you for your suggestions, we agree that these referenced articles will enhance the quality of the article.

Author Action: These article references have been added to the manuscript in Section 1.1. Please find these additions highlighted in green in the "Revised Manuscript with Track Changes" document.

9. **The outcomes of the study are not presented in the conclusion section. Specific conclusions are required.**

Author Response: Thank you for this comment, the article has been revised to highlight the conclusions more explicitly.

Author Action: Updates have been made to the conclusions section to better describe the variables, metrics, and their values within the discussion. Please see these changes highlighted in green in the “Revised Manuscript with Track Changes”.

Reviewer #2 Comment Responses

Reviewer #2: The presented article has potential, but in my view, it has some issues. The structure of the work is not adequate:

i) the introduction is long and should not contain figures or tables. The research question, objectives, and hypotheses should be evident. Part of what is in the review should go to a literature review chapter

Author Response: We agree that a dedicated Literature Review section would be beneficial as well as more explicit statements of the research questions, objectives, and hypotheses.

Author Action: A dedicated Literature Review section has been added and significant changes made to provide clarity. Please find these changes highlighted in green in the “Revised Manuscript with Track Changes”.

ii) the method is not clear. Statistical hypotheses are not defined, and independent or causal variables are not identified. The experiment design is not clearly explained. The estimates of the indicators are punctual. It would be more appropriate to consider the confidence intervals and statistical significance. For example, capability indices.

Author Response: Thank you for your comment. We recognize that the use of statistical tools is difficult in HMLV environments given the inherent variability and unstable data distributions.

Author Action: A description of the processes for statistical analysis (n=1) and an understanding of why this approach is necessary have been added to Section 3, footnote 1, and Appendix B has been added for additional detail. Please find these updates highlighted in green in the “Revised Manuscript with Track Changes”.

iii) The tables are presented far from the text, in an unordered way, and are not clear.

Author Response: We agree that this is a formatting challenge.

Author Action: We will support and provide feedback to the typesetting staff when it is typeset for final publication.

iv) The analyses should be based on the variables identified in the method section.

Author Response: Thank you for your comment. We agree this would further enhance the article.

Author Action: Values, variables, and metrics have been more explicitly identified within the text of the document to refer to those listed in the methods section. Please see these changes highlighted in green throughout the text in the “Revised Manuscript with Track Changes”.

v) The conclusions should adhere to the objectives and statistical hypotheses.

Author Response: We agree that a better description of the processes and, more specifically, the reasoning for the processes used would be helpful.

Author Action: Several updates have been made, which are highlighted in green in the “Revised Manuscript with Track Changes”. A significant change to the description of the processes used has been added to Section 5.
